# Complete genome sequence of T’Ho virus, a novel putative flavivirus from the Yucatan Peninsula of Mexico

**DOI:** 10.1186/s12985-017-0777-6

**Published:** 2017-06-12

**Authors:** Thomas Briese, Maria A. Loroño-Pino, Julian E. Garcia-Rejon, Jose A. Farfan-Ale, Carlos Machain-Williams, Karin S. Dorman, W. Ian Lipkin, Bradley J. Blitvich

**Affiliations:** 1 Center for Infection and Immunity, Mailman School of Public Health, Columbia University, New York, NY USA; 2 Laboratorio de Arbovirología, Centro de Investigaciones Regionales Dr. Hideyo Noguchi, Universidad Autónoma de Yucatán, Mérida, Yucatán Mexico; 3 Departments of Statistics and Genetics, Development and Cell Biology, College of Liberal Arts and Sciences and College Agriculture and Life Sciences, Iowa State University, Ames, IA USA; 4 Department of Veterinary Microbiology and Preventive Medicine, College of Veterinary Medicine, Iowa State University, Ames, IA 50011 USA

**Keywords:** Flavivirus, T’Ho virus, Genome sequence, High-throughput sequencing, Mexico, *Culex quinquefasciatus*

## Abstract

**Background:**

We previously reported the discovery of a novel, putative flavivirus designated T’Ho virus in *Culex quinquefasciatus* mosquitoes in the Yucatan Peninsula of Mexico. A 1358-nt region of the NS5 gene was amplified and sequenced but an isolate was not recovered.

**Results:**

The complete genome of T’Ho virus was sequenced using a combination of unbiased high-throughput sequencing, 5′ and 3′ rapid amplification of cDNA ends, reverse transcription-polymerase chain reaction and Sanger sequencing. The genome contains a single open reading frame of 10,284 nt which is flanked by 5′ and 3′ untranslated regions of 97 and 556-nt, respectively. Genome sequence alignments revealed that T’Ho virus is most closely related to Rocio virus (67.4% nucleotide identity) and Ilheus virus (65.9%), both of which belong to the Ntaya group, followed by other Ntaya group viruses (58.8–63.3%) and Japanese encephalitis group viruses (62.0–63.7%). Phylogenetic inference is in agreement with these findings.

**Conclusions:**

This study furthers our understanding of flavivirus genetics, phylogeny and diagnostics. Because the two closest known relatives of T’Ho virus are human pathogens, T’Ho virus could be an unrecognized cause of human disease. It is therefore important that future studies investigate the public health significance of this virus.

## Background

The genus *Flavivirus* (family *Flaviviridae*) contains more than 70 viruses, most of which are transmitted to vertebrates by arthropod vectors such as mosquitoes and ticks [23]. The genus is divided into at least 14 groups on the basis of nucleotide (nt) and deduced amino acid sequence data, antigenic relatedness and other characteristics. Two groups within the genus are the Ntaya and Japanese encephalitis (JE) groups. According to the Ninth Report of the International Committee on Taxonomy of Viruses, the Ntaya group consists of six viruses: Bagaza virus (BAGV), Ilheus virus (ILHV), Israel turkey meningoencephalitis virus (ITV), Ntaya virus (NTAV), Tembusu virus (TMUV) and Zika virus (ZIKV). Rocio virus (ROCV) is considered to represent a subtype of ILHV. The JE group consists of eight viruses: Cacipacore virus (CPCV), Japanese encephalitis virus (JEV), Koutango virus (KOUV), Murray Valley encephalitis virus (MVEV), St. Louis encephalitis virus (SLEV), Usutu virus (USUV), West Nile virus (WNV) and Yaounde virus (YAOV), in addition to Alfuy virus (ALFV) and Kunjin virus (KUNV) which are considered to represent subtypes of MVEV and WNV, respectively.

All flaviviruses possess a single-stranded, positive-sense RNA genome of approximately 11 kb [29]. The genome encodes a major open reading frame (ORF) that is flanked by 5′ and 3′ untranslated regions (UTRs) of ~100 and ~400–700 nt, respectively. The ORF encodes a polyprotein that is co- and post-translationally cleaved to generate three structural proteins, designated the capsid (C), premembrane/membrane (prM/M) and envelope (E) proteins, and at least seven nonstructural (NS) proteins in the gene order: 5′–C–prM (M)–E–NS1–NS2A–NS2B–NS3–NS4A–2K–NS4B–NS5–3′. Some viruses in the JE group utilize efficient −1 ribosomal frameshifting to produce a larger NS1-related protein (NS1’) [18, 33].

Previously, we provided evidence that a novel flavivirus (designated T’Ho virus) occurs in the Yucatan Peninsula of Mexico [16]. The putative virus was identified in a pool of *Culex quinquefasciatus* mosquitoes collected in 2007 at the Merida zoo, Yucatan State. A 1358-nt region of the NS5 gene was amplified and sequenced by reverse transcription-polymerase chain reaction (RT-PCR) and Sanger sequencing using flavivirus-specific primers. Application of BLASTn analysis revealed that the sequence is genetically equidistant to the corresponding regions of SLEV (72.6% identical), ILHV (72.2%), JEV (72.1%), USUV (71.8%), ROCV (71.4%), MVEV (71.3%), WNV (71.1%) and BAGV (70.1%). Although we successfully amplified T’Ho virus RNA, we were not able to obtain an isolate by virus isolation in African Green Monkey kidney (Vero) or *Aedes albopictus* (C6/36) mosquito cells or suckling mouse brain inoculation. In this study, the complete genome sequence of T’Ho virus was determined and its genetic relatedness to other flaviviruses was assessed.

## Methods

### High-throughput sequencing

Trizol Reagent (Invitrogen, Carlsbad, CA, USA) was used to extract total RNA from the pool of *Cx. quinquefasciatus* previously shown to contain T’Ho virus RNA. Protocols used for the collection, identification and homogenization of mosquitoes have been described elsewhere [16]. RNA extracts were reverse transcribed using SuperScript III (Thermo Fisher, Waltham, MA, USA) with random hexamers. The complementary DNA (cDNA) was RNase-H treated prior to second strand synthesis with Klenow Fragment (NEB, Ipswich, MA, USA). The generated double stranded (ds) DNA was sheared to an average fragment size of 200 bp using manufacturer’s standard settings (Covaris focused-ultrasonicator E210; Woburn, MA, USA). Sheared products were purified (Agencourt Ampure DNA purification beads, Beckman Coulter, Brea, CA, USA) and libraries constructed. Sheared nucleic acid was end-repaired, dA-tailed, ligated to sequencing adapters (NEBNext modules, NEB), PCR amplified (Phusion High-Fidelity DNA polymerase, NEB) and quantitated by Bioanalyzer (Agilent, Santa Clara, CA, USA) for sequencing. Sequencing on the Illumina HiSeq 2500 platform (Illumina, San Diego, CA, USA) resulted in an average of 180 million reads per lane. Samples were de-multiplexed using Illumina software and FastQ files generated. Data were quality filtered and trimmed (Slim-Filter) and de novo assembled using Dwight assembler at custom settings [20]. The generated contiguous sequences (contigs) and unique singleton reads were subjected to homology search using BLASTn and BLASTx against the GenBank database.

### 5′ and 3′ rapid amplification of cDNA ends

The extreme 5′ and 3′ ends of the T’Ho virus genome were determined by 5′ rapid amplification of cDNA ends (RACE) and 3′ RACE, respectively. In the 5′ RACE reactions, total RNA was reversed transcribed using a T’Ho virus-specific primer. Complementary DNAs were purified by ethanol precipitation and oligo (dC) tails were added to the 3′ ends using 15 units of terminal deoxynucleotidyl transferase (Invitrogen, Carlsbad, CA, USA). Tailing reactions were performed at 37 °C for 30 min and then terminated by heat-inactivation (65 °C for 10 min). Oligo dC-tailed cDNAs were purified by ethanol precipitation then PCR-amplified using a consensus forward primer specific to the C-tailed termini (5′-GACATCGAAAGGGGGGGGGGG-3′) and a reverse primer specific to the T’Ho virus cDNA sequence. In the 3′ RACE reactions, polyadenylate [poly (A)] tails were added to the 3′ ends of the T’Ho virus genomic RNA using 6 units of poly (A) polymerase (Ambion, Austin, TX, USA). Tailing reactions were performed at 37 °C for 1 h and then terminated by heat-inactivation (65 °C for 10 min). Poly (A)-tailed RNA was reverse transcribed using a poly (A) tail-specific primer (5′-GGCCACGCGTCGACTAGTACTTTTTTTTTTTTTTTTT-3′). Complementary DNAs were PCR amplified using a forward primer specific to the T’Ho virus cDNA sequence and a reverse primer that matched the 5′ half of the poly (A)-specific reverse transcription primer (5′-GGCCACGCGTCGACTAGTAC-3′). PCR products generated from the 5′ and 3′ RACE reactions were inserted into the pCR4-TOPO cloning vector (Invitrogen, Carlsbad, CA, USA) and ligated plasmids were transformed into competent TOPO10 *Escherichia coli* cells (Invitrogen, Carlsbad, CA). Cells were grown on Lysogeny broth (LB) agar containing ampicillin (50 μg/ml) and kanamycin (50 μg/ml), and colonies were screened for inserts by PCR amplification. An aliquot of each PCR product was examined by 1% agarose gel electrophoresis and several PCR products were purified using a QIAquick spin column (Qiagen, Valencia, CA, USA) and sequenced using a 3730 × 1 DNA sequencer (Applied Biosystems, Foster City, CA, USA).

### Nucleotide and amino acid sequence alignments

The genomic and predicted amino acid sequences of T’Ho virus were aligned to all other sequences in the Genbank database by application of BLASTn and BLASTp, respectively [1]. Sequence alignments using Clustal Omega (available at http://www.ebi.ac.uk/Tools/msa/clustalo/) were performed to calculate percent nucleotide and amino acid identities between select sequences.

### Phylogenetic analysis

The complete ORF sequences of T’Ho virus and other flaviviruses were aligned using Muscle v3.8.31 [14]. Phylogenetic trees were constructed by MrBayes v3.2.6 (evolutionary model GTR with gamma-distributed rates in six categories plus invariant sites, two chains run for 3 × 10^6^ generations with 25% burn-in, resulting in maximum PSRF 1.005 among all continuous parameters) [38], neighbor-joining (NJ, Mega v7.0.20 using Maximum Composite Likelihood distances with Gamma-distributed rates, alpha set to 0.58, pairwise gap deletion and 500 bootstraps), maximum parsimony (MP, Mega v7.0.20 using complete gap deletion and 500 bootstraps) [26] and maximum likelihood (ML, RaxML v8.2.8, using GTRGAMMAIX model and bootstopping criterion autoMRE) [42]. The GTR + G + I model was selected by sample size corrected AICc in jModelTest v2.1.6, and this finding motivated the models chosen for the NJ and ML analyses [36].

## Results

### Genomic organization and BLAST analysis

The T’Ho virus genome consists of 10,937 nt (Genbank Accession No. EU879061.2) and contains a single 10,284-nt ORF flanked by 5′ and 3′ UTRs of 97 and 556-nt, respectively. The ORF encodes the three structural and seven nonstructural proteins common to all known flaviviruses. The position and length of each gene and untranslated region are shown in Table 1. The complete genome and deduced polyprotein amino acid sequences of T’Ho virus were aligned to all other flaviviruses sequences for which complete genome data are available. The nucleotide sequence alignments indicated that T’Ho virus is most closely related to ROCV (67.4% identity) and ILHV (65.9%) followed by other Ntaya group viruses (58.8–63.3%) and JE group viruses (62.0–63.7%) (Table 2). The polyprotein of T’Ho virus has greatest amino acid identity to ROCV (72.2%) and ILHV (70.6%) followed by other Ntaya group viruses (57.1–66.3%) and JE group viruses (63.2–66.3%). The genome sequence of T’Ho virus was inspected for potential overlapping genes but none were identified.

**Table 1:** Genomic organization of T’Ho virus

Gene/genetic region	Size (nt)	Genome position	Protein size (aa)	Polyprotein position
5′ UTR	97	1–97	-	-
C	357	98–454	119	1–119
VirC	306	98–403	102	1–102
AnchC	51	404–454	17	103–199
prM	501	455–955	167	120–286
pr	276	455–730	92	120–211
M	225	731–955	75	212–286
E	1503	956–2458	501	287–787
NS1	1059	2459–3517	353	788–1141
NS2A	678	3518–4195	226	1142–1367
NS2B	393	4196–4588	131	1368–1498
NS3	1857	4589–6445	619	1499–2117
NS4A	378	6446–6823	126	2118–2243
2 K	69	6824–6892	23	2244–2266
NS4B	771	6893–7663	257	2267–2523
NS5	2718	7664–10,381	905	2524–3428
3′ UTR	556	10,382–10,937	-	-

**Table 2:** Genetic relatedness between T’Ho virus and select other flaviviruses

Virus	% nucleotide identity (genome)	% nucleotide identity (ORF)	% amino acid identity (polyprotein)
Ntaya group viruses			
Bagaza virus	62.6	63.5	66.0
Ilheus virus	65.9	65.8	70.6
^1^Rocio virus	67.4	67.3	72.2
Israel turkey meningoencephalitis virus	63.3	64.0	66.3
Ntaya virus	62.9	63.6	65.2
Tembusu virus	63.0	63.4	65.3
Zika virus	58.8	59.4	57.1
Japanese encephalitis group viruses			
Cacipacore virus	n/a	62.5	63.2
Japanese encephalitis virus	63.5	63.8	64.7
Koutango virus	n/a	63.4	64.0
Murray Valley encephalitis virus	62.8	63.3	64.4
^2^Alfuy virus	62.3	62.6	64.1
St. Louis encephalitis virus	63.7	64.1	66.3
Usutu virus	63.1	63.4	64.3
West Nile virus	62.8	63.4	64.5
^3^Kunjin virus	62.0	62.8	64.5
Yaounde virus	n/a	63.8	65.5
Other flaviviruses			
Cell fusing agent virus	42.1	42.0	29.2
Dengue virus type-2	56.4	56.9	51.7
Modoc virus	50.2	48.1	38.4
Tick-borne encephalitis virus	48.5	49.1	41.9
Yellow fever virus	53.4	53.7	45.8

^1, 2, 3^Subtype of ILHV, MVEV and WNV, respectively

*n/a* not available (genome has not been fully sequenced)

### Organization of the polyprotein

Cleavage sites in the T’Ho virus polyprotein are shown in Table 3. These sites were predicted by aligning the polyprotein sequence of T’Ho virus to those of select other flaviviruses for which cleavage sites have previously been predicted and in some instances experimentally verified. The predicted cleavage sites for T’Ho virus are in agreement with the rules previously established for flaviviruses. The C/prM, prM/E, E/NS1 and 2K/NS4B polyprotein junctions of most known flaviviruses conform to predicted signalase cleavage sites [6] and similar sites were identified at these locations for T’Ho virus (Table 3). The NS1/NS2A junctions of most known flaviviruses occur after a Val-X-Ala site that fulfills the ‘-1, −3’ rule for a signalase site but lacks an upstream hydrophobic domain [6]. The predicted NS1/NS2A junction of T’Ho virus fulfills these requirements. For most flaviviruses, the predicted pr/M junction occurs after an Arg-X-Lys/Arg-Arg or Arg-X-X-Arg motif. T’Ho virus adheres to this rule. The flavivirus virion C/anchor, NS2A/NS2B, NS2B/NS3, NS3/NS4A, NS4A/2K and NS4B/NS5 junctions are commonly cleaved after two basic amino acid residues (KR, RR or RK) [5–7] and sites in T’Ho virus are consistent with that rule.

**Table 3:** Predicted cleavage sites in the polyproteins of T’Ho virus and select other flaviviruses

Virus	Junction					
	^1^VirC/Anch	^2^C/prM	^3^pr/M	^2^prM/E	^2^E/NS1	^4^NS1/NS2A
T’Ho virus	KNNKR↓GMTTT	ATTMA↓ARLSS	RRGRR↓SINIA	VPAYS↓LNCLG	MNVHA↓DTGCV	SMVVA↓GHGSS
ROCV	RKAKR↓GNGSV	TGSMA↓LRLGT	RRGRR↓SVNIP	APAYS↓INCLG	MNVHA↓DTGCA	SKVTA↓GTGND
ILHV	KEKKK↓SFSTA	TAVAG↓LKISS	RRGRR↓AINIP	APAYS↓LNCLG	VNVHA↓DTGCA	SKVSA↓GNGQT
BAGV	GKKKR↓GGTTV	GVAQA↓IKIGS	RRSRR↓SITVH	APAYS↓FNCLG	TNVHA↓DTGCA	SRVTA↓YDGAG
WNV	KQKKR↓GGTAG	ACAGA↓VTLSN	RRSRR↓SLTVQ	APAYS↓FNCLG	VNVHA↓DTGCA	SRVNA↓YNADM
	^1^NS2A/NS2B	^1^NS2B/NS3	^1^NS3/NS4A	^1^NS4A/2K	^2^2K/NS4B	^1^NS4B/NS5
T’Ho virus	GMTRR↓GWPAS	RLQKR↓GGVLW	AAGKR↓SASAI	PEKQR↓SQTDN	SAVSA↓NEMGW	PKVKR↓GGGTG
ROCV	CATKR↓GWPAS	KIHKR↓GGVLW	AAGKR↓SAGSM	PEKQR↓SQTDN	SAVSA↓NEMGW	PKVKR↓GGIAA
ILHV	SLGKR↓GWPAS	KIHKR↓GGVMW	AAGKR↓SAGSV	PEKQR↓SQTDN	GAVSA↓NEMGW	PKLKR↓GGGSA
BAGV	PSNRR↓GWPVS	HSPKR↓SGAIW	ACGKR↓SAIGV	PEKQR↓SQTDS	GTVAS↓NEMGW	GSMRR↓GGGKG
WNV	PNRKR↓GWPAT	QYTKR↓GGVLW	ASGKR↓SQIGL	PEKQR↓SQTDN	GAVAA↓NEMGW	PGLKR↓GGAKG

Predicted cleavage sites for ROCV, WNV and BAGV were identified in earlier studies ([8]; [6]; [27]; [32]). Most of the predicted cleavage sites for ILHV are described in the corresponding Genbank entry (accession no. AAV34155) and the remainder determined here. Underlined sequences have been experimentally verified. Cleavage events are mediated by ^1^the viral NS2B/NS3 serine protease ([6]; [7]; [5]), ^2^a host signal peptidase [6], ^3^the cellular furin protease ([6]; [35]; [41]) and ^4^a membrane-bound host protease in the endoplasmic reticulum [15].

Potential N-linked glycosylation sites were identified using the NetNGlyc 1.0 Server (available at http://www.cbs.dtu.dk/services/NetNGlyc/) with the consensus sequence defined as Asn-X-Ser/Thr (where X is not Pro) in the context of specific surrounding sequences. Fourteen Asn-X-Ser/Thr motifs were identified in the T’Ho virus polyprotein sequence. Eight motifs are predicted to be utilized by N-linked glycans: these are located at prM_15_, prM_148_, E_154_, NS1_130_, NS1_207_, NS2A_122_, NS5_234_ and NS5_655_.

### Phylogenetic analysis

**Fig. 1:**
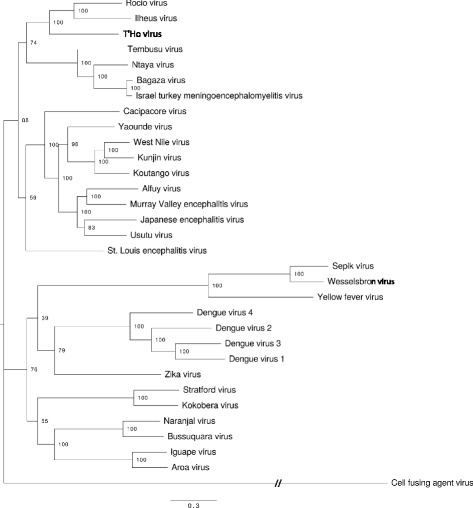
Unrooted maximum likelihood phylogeny of T'Ho virus and other relevant flaviviruses estimated by RAxML. The support out of 100 bootstraps is indicated on each branch. The branch length of cell fusing agent virus (CFAV; the outgroup) is not shown to scale: the actual estimated branch length is 15.60. Although T’Ho virus clusters with Ntaya and JE group viruses with 88% bootstrap support in this phylogeny and 98% posterior support in the MrBayes tree, the CFAV outgroup splits these two groups in around 50% of neighbor-joining bootstrap trees; there is considerable uncertainty in the deep branches of the phylogeny. Genbank Accession numbers for sequences used in the analysis are as follows: T’Ho virus, EU879061.2; Alfuy virus, AY898809.1; Aroa virus, KF917535.1; Bagaza virus, NC_012534.1; Bussuquara virus, AY632536.4; Cacipacore virus, LN849009.1; Cell fusing agent virus, NC_001564.1; Dengue virus type 1, AY277665.2; Dengue virus type 4, KF041260.1; Dengue virus type 2, U87411.1; Dengue virus type 3, AY099336.1; Iguape virus, AY632538.4; Ilheus virus, NC_009028.2; Israel turkey meningoencephalomyelitis virus, KC734552.1; Japanese encephalitis virus, NC_001437.1; Kokobera virus, NC_009029.2; Koutango virus, EU082200.2; Kunjin virus, KX394383.1; Murray Valley encephalitis virus, AF161266.1; Naranjal virus, KF917538.1; Ntaya virus, NC_018705.3; Rocio virus, AY632542.4; Sepik virus, NC_008719.1; St. Louis encephalitis virus, DQ525916.1; Stratford virus, KM225263.1; Tembusu virus, NC_015843.2; Usutu virus, NC_006551.1; Wesselsbron virus, NC_012735.1; West Nile virus, M12294.2; Yaounde virus, EU082199.2; Yellow fever virus, NC_002031.1 and Zika virus, NC_012532.1

A ML phylogenetic tree was constructed by RAxML v8.2.8 using the complete ORF sequences of T’Ho virus and all 30 mosquito-borne flaviviruses (species and subtypes) listed in the Ninth Report of the International Committee on Taxonomy of Viruses (Fig. 1). Cell fusing agent virus (an insect-specific flavivirus) was used as the outgroup. Phylogenetic trees were also constructed using Bayesian, NJ and MP methods (data not shown). In all trees, T’Ho virus was most closely related to ROCV and ILHV. The Bayesian posterior support for this grouping was strong (100%), in agreement with similarly strong bootstrap support (100% for ML, 100% for MP, 94% for NJ). These viruses cluster within a larger clade that contains the Ntaya group viruses with the exception of ZIKV, although in phylogenies based on short conserved polymerase motifs used in taxonomic classification ZIKV appears more closely related to the Ntaya group viruses [24]. The Bayesian posterior support for this topological arrangement is 100%, but bootstrap support is weaker ranging from 49% for MP to 79% for NJ. Trees generated by all methods agree on the composition of all clades shown in Fig. 1 with 98% or more bootstrap support, but the relative placement of these clades is uncertain (data not shown).

## Discussion

We report the complete genome sequence of T’Ho virus, a novel putative flavivirus discovered in *Cx. quinquefasciatus* from the Yucatan Peninsula of Mexico. The closest known relatives of T’Ho virus are ROCV and ILHV, that both belong to the Ntaya group of mosquito-borne flaviviruses, with 67.4% and 65.9% nt. identity, respectively. Based on genetic criteria, we propose that T’Ho virus is classified as a new species within the genus *Flavivirus*. It has been suggested that flaviviruses with >84% nucleotide sequence identity should be classified within the same species [28]. The genome of T’Ho virus has only 58.8–67.4% nt. identity (mean: 63.4%) to the seven viral species and subtypes currently assigned to the Ntaya group, and this amount of genetic relatedness is not dissimilar to that observed between group members (≥57.9% identity; mean: 65.9%). Phylogenetic analysis of full polyprotein sequences revealed that T’Ho virus forms a distinct clade with Ntaya group viruses, except for ZIKV. Based on previous sequence analysis of a 1358-nt region of the NS5 gene of T’Ho virus that indicated it to be genetically equidistant to ILHV, SLEV, WNV, ROCV and JEV, we speculated that T’Ho virus could also serologically resembled WNV [16]; however, the NS5 gene is one of the most conserved regions of the flavivirus genome [6] and sequence alignments and phylogenetic studies performed using relatively short, conserved sequences are not as robust as those performed with complete genomes.

The two closest known relatives of T’Ho virus are recognized human pathogens. ROCV was responsible for several epidemics of severe encephalitis in Brazil in the 1970s [10, 11, 17, 44]. A case fatality rate of 10% and long-term sequelae in 20% of the surviving patients were reported. ILHV has been sporadically isolated from humans in Central America, South America and the Caribbean, and symptomatic infections are usually characterized by fever, headache, myalgia and arthralgia although central nervous system manifestations have also been reported [25, 37, 39, 40, 45]. Because T’Ho virus is most closely related to known human pathogens, it too could be a cause of human disease. Most other Ntaya group viruses are also recognized pathogens; ZIKV is a cause of febrile illness, neonatal microcephaly and linked to Guillain-Barré syndrome [47]. BAGV, ITV and TMUV are known avian pathogens [4, 9, 30]. Most JE group viruses are also serious pathogens of humans and other vertebrates [21].

An isolate of T’Ho virus is not available; thus, experimental infection studies cannot currently be performed to assess the vector and reservoir competence of mosquito and vertebrate species likely to be involved in its amplification. However, probable vectors and reservoir hosts of T’Ho virus can be inferred from the information available for its closest known relatives. The amplification cycles of ROCV and ILHV are not well defined but most isolations have been made from *Aedes* and *Psorophora* spp. mosquitoes (particularly *Ps. ferox*) with birds implicated as principal reservoir hosts [2, 12, 17, 19, 31, 34]. Most other Ntaya group viruses are primarily maintained in transmission cycles between *Culex* spp. mosquitoes and birds [13, 22, 43], the notable exception being ZIKV which cycles between *Aedes* spp. mosquitoes and primates [47]. JE group viruses are primarily maintained in transmission cycles between *Culex* spp. mosquitoes and birds [21]. It is therefore likely that the principal amplification vectors of T’Ho virus are *Culex*, *Aedes* and/or *Psorophora* spp. mosquitoes and the principal reservoir hosts are birds.

## Conclusion

In conclusion, we describe a novel species, T’Ho virus, of the genus *Flavivirus*, whose closest known relatives are human pathogens; thus, it is feasible to suggest that T’Ho virus may be an unrecognized cause of human disease in the Yucatan Peninsula of Mexico. Our report enables directly the creation of specific PCR diagnostic assays. However, serological cross-reactivity is common with flaviviruses [3], as exemplified in the ongoing ZIKV pandemic where the differential diagnosis of ZIKV and dengue virus infections is difficult [46]. Accordingly, virus isolates or a recombinant virus will be needed for serosurveillance studies in Latin America where several other flaviviruses, including dengue virus, yellow fever virus, ZIKV, WNV, ROCV and ILHV, may co-circulate in overlapping geographic areas.

## Abbreviations

ALFV: Alfuy virus
BAGV: Bagaza virus
C: Capsid
cDNA: Complementary DNA
CPCV: Cacipacore virus
ds: Double stranded
E: Envelope
ILHV: Ilheus virus
ITV: Israel turkey meningoencephalitis virus
JE: Japanese encephalitis
JEV: Japanese encephalitis virus
KOUV: Koutango virus
KUNV: Kunjin virus
LB: Lysogeny broth
ML: Maximum likelihood
MP: Maximum parsimony
MVEV: Murray Valley encephalitis virus
NJ: Neighbor-joining
NS: Nonstructural
nt: Nucleotide
NTAV: Ntaya virus
ORF: Open reading frame
Poly (A): Polyadenylate
prM/M: Premembrane/membrane
RACE: Rapid amplification of cDNA ends
ROCV: Rocio virus
RT-PCR: Reverse transcription-polymerase chain reaction
SLEV: St. Louis encephalitis virus
TMUV: Tembusu virus
USUV: Usutu virus
UTRs: Untranslated regions
WNV: West Nile virus
YAOV: Yaounde virus
ZIKV: Zika virus
